# Quality management program in epithelial ovarian cancer: proposal in a Latin American country

**DOI:** 10.1136/ijgc-2022-003899

**Published:** 2022-11-11

**Authors:** Daniel Sanabria, Maria Camila Fernández, Natalia Hurtado, Adriana Ramos, Juliana Rodriguez

**Affiliations:** 1 Department of Gynecology, Obstetrics and Human Reproduction, Section of Gynecologic Oncology, Fundacion Santa Fe de Bogota, Bogota, Colombia; 2 Department of Gynecology and Obstetrics, Universidad El Bosque, Bogota, Colombia

**Keywords:** Ovarian Cancer

Ovarian cancer is the most lethal gynecological neoplasm and the ninth most common in women. Worldwide, by 2020, 313 959 cases had been diagnosed, and 207 252 women had died from this disease. For Latin America and the Caribbean, age-adjusted incidence and mortality rates of 14.9 and 7.6 per 100 000 women per year were reported, respectively.^1^ Approximately 75% of patients will have advanced disease at diagnosis. When possible, the standard treatment is primary cytoreductive surgery followed by platinum-based cytotoxic chemotherapy. The use of maintenance therapies has also been suggested in different clinical settings.^2^ Complete tumor resection has proven to be the most important prognostic factor for survival in these patients.^3^

Quality of surgical care is a major component of the multidisciplinary management of the disease.^4^ Implementation of a quality program allows for reduction in morbidity and costs.^5^ Different organizations promote improvement in the care of ovarian neoplasms. For instance, the European Society for Gynaecological Oncology (ESGO) certification for advanced ovarian cancer surgery is an award attributed to institutions which can offer patients the specific skills, experience, organization, and dedication that are required to achieve optimal levels of surgical care. The ESGO certification is based on the completion of 10 quality indicators and a scoring system.^6^ Currently, there are more than 60 accredited centers in Europe and Asia.^7^

In Latin America, non-medical barriers to cytoreductive surgery have been identified: low surgeon expertise, limitations on access, and/or inadequate resources.^8^ In Colombia, care is predominantly decentralized. There are no centers of excellence in ovarian cancer. In 2018, the project “Center for clinical care in epithelial ovarian cancer” was created at the Fundacion Santa Fe de Bogota, a private institution. The objectives of the center are to form an interdisciplinary working group specialized in the comprehensive management of epithelial ovarian cancer, to create protocols for diagnosis, treatment and follow-up, to meet international quality indicators, and to reduce costs associated with treatment. The inclusion criteria are: patients older than 18 years and younger than 75 years, histopathological confirmation of epithelial cancer of the ovary and/or fallopian tube, and stages I/IVA susceptible to management of cytoreductive surgery and intravenous chemotherapy.

**Figure 1:**
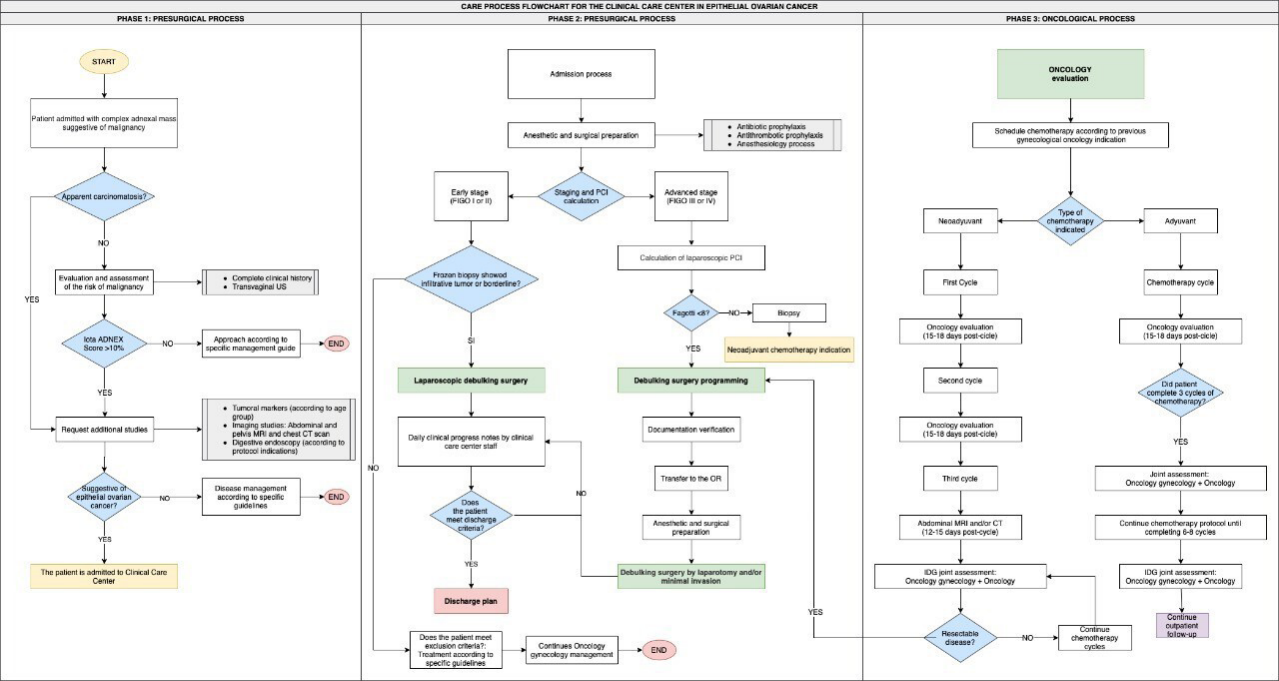
Flowchart of the process of the clinical care center

**Figure 2:**
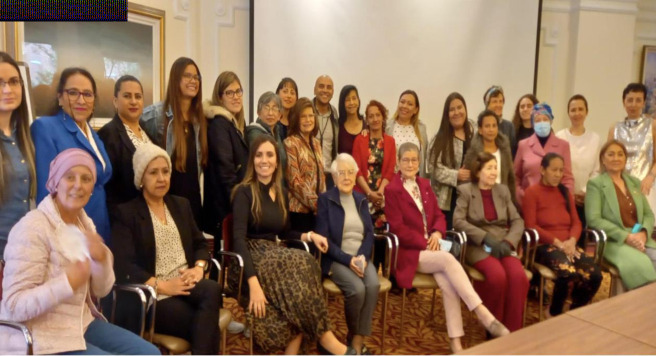
Patient symposium, May 2022.

The center has four phases of the process: diagnosis, surgical management, clinical oncologic process, and follow-up (Figure 1). A standardized protocol has been designed for each of these phases. There is the participation of a multidisciplinary team made up of 20 specialties, state-of-the-art technology, and infrastructure. The center has quality indicators adapted and aligned with international standards^9^ (Table 1). Active work has also been done on educating women and strengthening their support networks, through patient-focused symposiums (Figure 2). Currently this program is in the self-evaluation phase and is waiting to complete the accreditation by the Joint Commission International.

**Table 1:** Quality indicators in the ovarian cancer program

Quality indicators	Goal
*Patient care*	
Proportion of patients with complete surgical resection in upfront debulking or interval debulking surgery	>65%
Proportion of patients receiving antibiotic prophylaxis 60 min before starting cytoreductive surgery	100%
Proportion of patients receiving pharmacological anti-thrombotic prophylaxis within 24 hours of cytoreduction	100%
Proportion of patients who presented operative site infection during the 30 days after the procedure	<5%
Proportion of patients who start adjuvant chemotherapy within 42 days after debulking surgery	100%
Proportion of patients who completed the EORTC-QLQC30 quality of life questionnaire before starting cancer treatment	100%
Proportion of patients who completed the EORTC-QLQC30 quality of life questionnaire after completing cancer treatment	100%
*Infrastructure*	
Number of cytoreductive surgeries performed per surgeon per year	>100 Minimum required: >20
Surgeries supervised or performed by surgeons operating on at least 20 patients a year	≥95%
Rate of primary debulking surgeries	≥50%
Surgery performed by a gynecologic oncologist specifically dedicated to gynecological cancer management	Yes
Center participating in clinical trials in gynecologic oncology	Yes
Treatment planned and reviewed at a multidisciplinary team meeting	≥90%
Required pre-operative workup	≥95%
Pre-operative, intra-operative, and post-operative management	Yes
Minimum required elements in operative reports	90%
Minimum required elements in pathology reports	>90%
Structured prospective reporting of post-operative complications	100%

Adapted from Fotopolou et al.^9^ Quality indicators for advanced ovarian cancer surgery from the European Society of Gynaecological Oncology (ESGO): 2020 update. *Int J Gynecol Cancer* 2020;30:436–40.

Ovarian cancer care is challenging, especially in developing countries. The introduction of quality programs and management centralization of this condition will improve not only care, but clinically relevant outcomes in our population.
